# Mandibular Distraction Osteogenesis for Upper Airway Obstruction in Infants and Neonates: Airway Outcomes, Hospitalization Burden and Procedure Application in the Polish Population

**DOI:** 10.3390/jcm15208109

**Published:** 2026-10-19

**Authors:** Patryk Kołodziejski, Aleksandra Kołodziejska, Sebastian Radziak, Anna Krzykowska, Barbora Hocková, Krzysztof Dowgierd

**Affiliations:** 1 Department of Maxillofacial and Reconstructive Surgery for Children and Adolescents, Regional Specialized Children’s Hospital, 18a Zolnierska Str., 10-561 Olsztyn, Poland; 2 Department of Conservative Dentistry, Faculty of Medicine, Medical University of Gdańsk, 80-210 Gdansk, Poland; 3 Department of Maxillofacial Surgery, Faculty Hospital F. D. Roosevelt, Slovak Medical University, 975 17 Banská Bystrica, Slovakia; 4 Head and Neck Surgery Clinic for Children and Young Adults, Department of Clinical Pediatrics, University of Warmia and Mazury in Olsztyn, Regional Specialized Children’s Hospital, 18a Zolnierska Str., 10-561 Olsztyn, Poland

**Keywords:** Robin sequence, distraction osteogenesis, mandibular hypoplasia, infant, airway obstruction

## Abstract

**Objectives**: This study retrospectively evaluated the respiratory outcomes and hospitalization burden of infants and neonates diagnosed with upper airway obstruction who underwent bilateral mandibular distraction osteogenesis in the Polish population. **Methods**: The study analyzed 40 patients aged 0–12 months who underwent bilateral MDO for respiratory insufficiency associated with mandibular hypoplasia between 2013 and 2025. Successful airway management was defined as tracheostomy avoidance, without the need for further respiratory support in non-tracheostomized patients, or successful decannulation, with no requirement for additional respiratory support in patients with pre-existing tracheostomy. Duration of hospitalization and intensive care unit stay were also evaluated. **Results**: Twenty three patients had iPRS and 17 had sPRS. An overall successful airway outcome was achieved in 30/40 patients (75.0%), with a significantly higher success rate in iPRS than sPRS (95.7% vs. 47.1%, *p* < 0.001). MDO prevented tracheostomy in 28/30 (93.3%) patients, including all 20 patients with iPRS. In contrast, only 2/10 (20.0%) previously tracheostomized patients were successfully decannulated, both with iPRS. Patients with successful airway outcomes were younger at MDO (median 54 vs. 138 days, *p* = 0.015). The multivariable analysis indicated sPRS as an independent predictor of respiratory failure after adjustment for age. **Conclusions**: Bilateral MDO provided effective airway management, particularly in patients with iPRS, and was highly effective in avoiding a new tracheostomy. Syndromic diagnosis and pre-existing tracheostomy characterized patients with substantially less favorable airway outcomes, highlighting clinically important differences within this heterogeneous population.

## 1. Introduction

Micrognathia is an important potential cause of upper airway obstruction and respiratory insufficiency in neonates. It may be an isolated craniofacial anomaly or occur as a component of an underlying congenital syndrome. Pierre Robin sequence (PRS) is the most common condition associated with micrognathia and consequent respiratory compromise. Other conditions characterized by micrognathia include Treacher Collins syndrome, Stickler syndrome and Nager syndrome.

The PRS was first described by the French physician Pierre Robin in 1923 as a clinical triad: micrognathia, glossoptosis and upper airway obstruction. In 1934 the original definition was modified and expanded by the author by adding a U-shaped cleft palate [1]. These characteristic features may occur as an isolated craniofacial anomaly or in association with an underlying genetic syndrome. The prevalence of PRS varies from one in 8080 births in Germany to one in 14,000 births in Denmark [2,3]. In a recent review based on EUROCAT registries, the estimated incidence is approximately 12 per 100,000 births for all PRS cases and 7.8 per 100,000 births for isolated PRS [4].

In the present study, we distinguish isolated PRS (iPRS) and syndromic PRS (sPRS), both of which may be associated with neonatal respiratory insufficiency. Although the characteristic craniofacial abnormalities may be limited to an aesthetic manifestation in some patients, more severe phenotypes can result in significant upper airway obstruction and potentially life-threatening respiratory failure. PRS may also be associated with feeding difficulties and failure to thrive, potentially resulting from the increased work of breathing and respiratory effort [5,6,7].

The majority of patients do not require surgical treatment and their symptoms can be managed with non-invasive techniques [8,9]. These include prone or lateral positioning, a nasopharyngeal airway and continuous positive airway pressure (CPAP). Surgical intervention may be indicated in patients with persistent respiratory symptoms despite conservative management or in those with severe upper airway obstruction. A variety of surgical approaches have been developed over the years, with tongue–lip adhesion (TLA) and mandibular distraction osteogenesis (MDO) currently representing the principal surgical treatment modalities. In selected cases, subperiosteal release of the floor of the mouth may be considered.

Distraction osteogenesis is an established surgical technique for the correction of skeletal deficiencies and discrepancies. It is based on the controlled, gradual elongation of bone following the application of mechanical traction. Its biological effects extend beyond the osseous structures to the surrounding soft tissues and adjacent anatomical structures [10]. It was first introduced in orthopedic surgery by Codivilla [11] and Ilizarov [12], who laid the foundation for long bone distraction. MDO was introduced into craniofacial surgery by McCarthy in 1992 [13], with external distractor use. Subsequently, Denny et al. reported the use of MDO for the treatment of airway obstruction in neonates with the Pierre Robin sequence in 2004 [14].

Since its introduction, bilateral mandibular distraction osteogenesis (BMDO) has become an established surgical approach for the management of severe upper airway obstruction associated with mandibular hypoplasia in newborns and neonates. Over the past two decades, substantial advances have been made in both surgical techniques and distraction-device technology, contributing to the refinement of treatment protocols and clinical outcomes.

In the present study, we report our institutional experience with BMDO in infants and neonates treated at a specialized craniofacial center in Poland—the only referral center in Poland where BMDO for infants and neonates is performed. The aim of this study was to evaluate airway outcomes following BMDO in neonates and infants with iPRS and sPRS. Moreover the study evaluates hospitalization burden, analyzes potential predictors of failure and describes the use of MDO in the Polish population.

## 2. Materials and Methods

### 2.1. Data Collection

This retrospective cohort study adhered to the Strengthening the Reporting of Observational Studies in Epidemiology (STROBE) reporting guidelines. Ethical approval was granted by the Institutional Review Board of the Regional Medical Council of Warmia and Mazury, Poland (L.Dz.WMIL-KB/67/2025; 4 September 2025). The study was conducted in compliance with the principles of the Declaration of Helsinki (59th revision, 21 October 2008). The study population comprised newborns and infants with upper airway obstruction and respiratory insufficiency who were treated at the Regional Specialist Children’s Hospital in Olsztyn (a tertiary referral center in Poland) between 1 January 2013 and 1 September 2025.

Patient identification was performed through a systematic search of the institutional electronic medical database using the following International Statistical Classification of Diseases and Related Health Problems, 10th Revision (ICD-10) codes: P28.4; P28.5; J96.0; J96.9; Q87.0; Q87.8; Q75.4. The search was conducted between 8 May and 26 May 2026, and identified 123 potentially eligible patients.

Additionally, a manual review of the hospital archives (3–15 June 2026) yielded an additional 20 cases. After application of the predefined eligibility criteria, 103 cases were excluded, yielding a final cohort of 40 patients.

Clinical and demographic data were independently collected by two surgeons, each with at least five years of clinical experience. An independent review of all included cases was subsequently performed by a senior maxillofacial surgeon to verify eligibility and ensure the accuracy and completeness of the collected data.

The study included patients aged 0–12 months with clinically confirmed upper airway obstruction and respiratory insufficiency attributable to mandibular hypoplasia who underwent BMDO at the study institution. All patients included in the analysis underwent postoperative follow-up for a minimum of 12 months. Patients were excluded in the presence of incomplete medical documentation, unilateral mandibular distraction, unilateral mandibular hypoplasia, or temporomandibular joint ankylosis.

The variables extracted from the medical records included demographic characteristics, underlying clinical diagnosis, anatomical site of mandibular involvement, age at the time of surgery and time of hospital and ICU stay. Operative records were systematically reviewed to document the type of distraction device, activator placement, and the degree of intraoperative distraction.

### 2.2. Preoperative Patient Evaluation

Children with micrognathia and persistent respiratory insufficiency despite conservative management, including prone positioning, supplemental oxygen delivered via nasal cannula and nasogastric feeding, underwent a comprehensive multidisciplinary evaluation. The multidisciplinary team consisted of specialists in otorhinolaryngology, pediatrics, anesthesiology, neonatology and maxillofacial surgery. The diagnostic assessment included computed tomography (CT) imaging to delineate the craniofacial skeletal anatomy and airway endoscopy to assess the upper and lower airway and exclude conditions such as tracheomalacia and subglottic stenosis. When clinically appropriate, polysomnography (PSG) was performed to characterize the nature of apneic events and objectively determine the severity of respiratory impairment. Patients demonstrating central apnea on polysomnography, evidence of tracheomalacia or subglottic stenosis were not considered as candidates for BMDO. In contrast, patients with severe mandibular hypoplasia requiring airway instrumentation or tracheostomy were regarded as potential candidates for surgical intervention. Eligibility was also considered in patients with apnea refractory to conservative management and signs of obstructive apnea in PSG such as an apnea-hypopnea index (AHI) > 5 events/h. The final decision regarding BMDO was established through multidisciplinary consensus in all cases.

### 2.3. Surgical Technique

The surgical procedures were performed with intraoperative nerve stimulation to facilitate identification and preservation of the marginal mandibular branch of the facial nerve. The mandibular body was accessed through a submandibular Risdon incision followed by identification of the mandibular angle and retromolar triangle. A KLS Martin (Tuttlingen, Germany) neonatal distraction device was subsequently adapted to the mandible and secured with screws. A C-shaped osteotomy was performed using a piezoelectric saw. Attention was paid to minimize the risk of injury to the inferior alveolar nerve and tooth buds. Following osteotomy, correct positioning and functionality of the distraction device were verified by controlled activation. The distractor was subsequently returned to its initial position. The activation rods were exteriorized through the skin via either the retromandibular or submandibular region to allow postoperative activation.

Following surgery, patients were transferred to the Intensive Care Unit (ICU), where distractor activation was initiated after a one-day latency period. The activation protocol consisted of one turn of the device (0.3 mm) three times daily.

Extubation was performed after initial improvement in airway patency. Subsequent distraction was continued in the Neonatal Unit until 2–3 mm of anterior overjet was achieved. The effectiveness of BMDO was defined by tracheostomy avoidance, without the need for further respiratory support in non-tracheostomized patients (assessed at the end of the activation phase), or successful decannulation, with no requirement for additional respiratory support in patients with pre-existing tracheostomy (decannulation within 12 months of surgery).

### 2.4. Follow-Up

All patients included in the study underwent postoperative follow-up at the study institution for a minimum of 12 months. The initial outpatient assessment was scheduled approximately one week after hospital discharge. During this visit, the activation rods were removed when clinically feasible. Subsequent outpatient assessments were scheduled according to individual clinical needs and postoperative courses. A consolidation period of at least 3 months was maintained before planned removal of the distractor device.

### 2.5. Data Analysis

Forty patients were included in the final analysis. Demographic characteristics, treatment modalities and clinical outcomes were analyzed using descriptive statistical methods. The distribution of continuous variables was assessed using the Shapiro–Wilk test. Depending on the distribution of the data, continuous variables were presented as mean ± standard deviation (SD) or median with interquartile range (IQR), as appropriate. Categorical variables were expressed as absolute frequencies or percentages.

Comparisons between study groups were performed using Fisher’s exact test for categorical variables and the Mann–Whitney U test for continuous variables. Spearman’s rank correlation coefficient was calculated to estimate the relationships of age with hospitalization length and postoperative ICU stay.

To identify independent predictors of respiratory outcome after mandibular distraction osteogenesis (MDO), multivariable logistic regression analysis was performed using Firth’s penalized likelihood method. Candidate predictors were restricted to variables available before or at the time of MDO. They were selected on the basis of clinical relevance and univariable association with respiratory outcome. Variables demonstrating a potential association at *p* < 0.20 in univariable analysis were considered for multivariable modeling. Adjusted odds ratios (aORs) with 95% confidence intervals (CIs) and corresponding *p*-values were reported.

All statistical analyses were conducted using jamovi statistical software (version 2.7.38) (Sydney, Australia), an open-source statistical platform. All tests were two-sided, and the level of statistical significance was set at *p* < 0.05.

## 3. Results

### 3.1. Demographic Findings

A total of 40 children who underwent surgical intervention for upper airway obstruction were included in the study cohort. The cohort comprised 25 males and 15 females. The mean age at the time of MDO was 87.0 (±76.7 days). The youngest patient underwent the procedure at 14 days of age. Patients with iPRS were significantly younger at the time of surgery than patients with sPRS: median 52 days (IQR 35–63) versus 97 days (IQR 43–153), respectively (*p* = 0.030). The corresponding mean ages were 64.9 ± 62.5 and 116.9 ± 85.5 days. A history of prematurity was documented in 10 patients within the study cohort.

**Figure 1:**
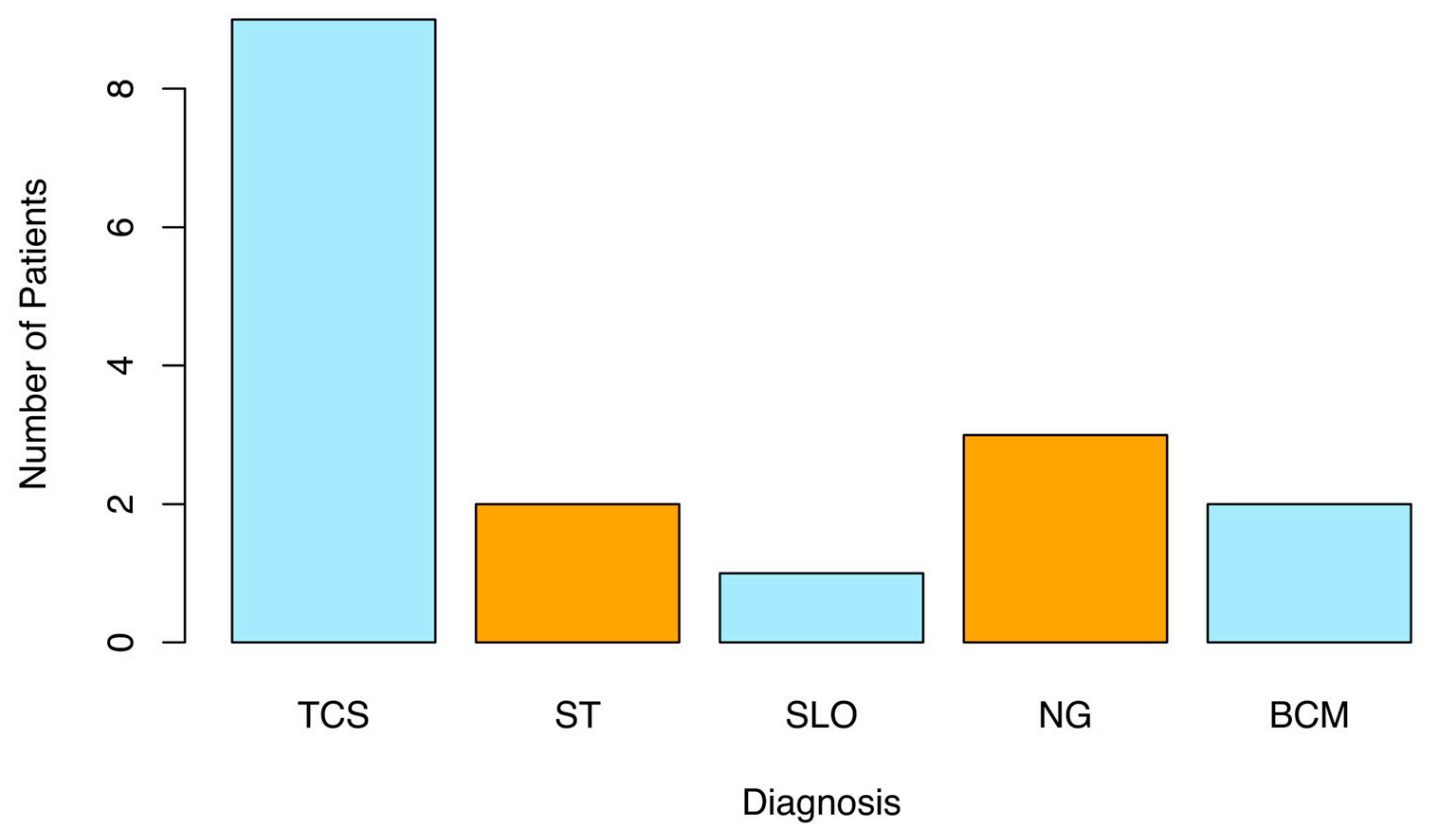
Syndromes diagnosed in sPRS group. TCS—Treacher Collins syndrome; NG—Nager syndrome; BCM—bilateral craniofacial microsomia; ST—Stickler syndrome; SLO—Smith–Lemli–Opitz Syndrome.

Twenty-three patients were diagnosed with iPRS, whereas 17 had sPRS. Within the sPRS subgroup, Treacher Collins syndrome was the most frequently identified diagnosis (9/40), followed by Nager syndrome (3/40), bilateral craniofacial microsomia (2/40), Stickler syndrome (2/40) and Smith–Lemli–Opitz (1/40). Cleft palate was observed in 35 patients (87.5%). Detailed clinical characteristics of the sPRS subgroup are presented in Figure 1.

### 3.2. BMDO Distribution in Poland

Forty BMDO procedures were performed between 2013 and 2025 in our center. At least one procedure was performed in each year of the analyzed period. The mean annual number of BMDO procedures was 3.08 ± 2.02, with a median of two procedures per year between 2013 and 2025.

Gradually the annual number of procedures was increasing. Eight procedures were performed between 2013 and 2016, corresponding to a mean of 2.0 procedures per year. Between 2017 and 2020, the number increased to 10 procedures (mean, 2.5 per year), while 22 procedures were performed between 2021 and 2025 (mean, 4.4 per year).

**Figure 2:**
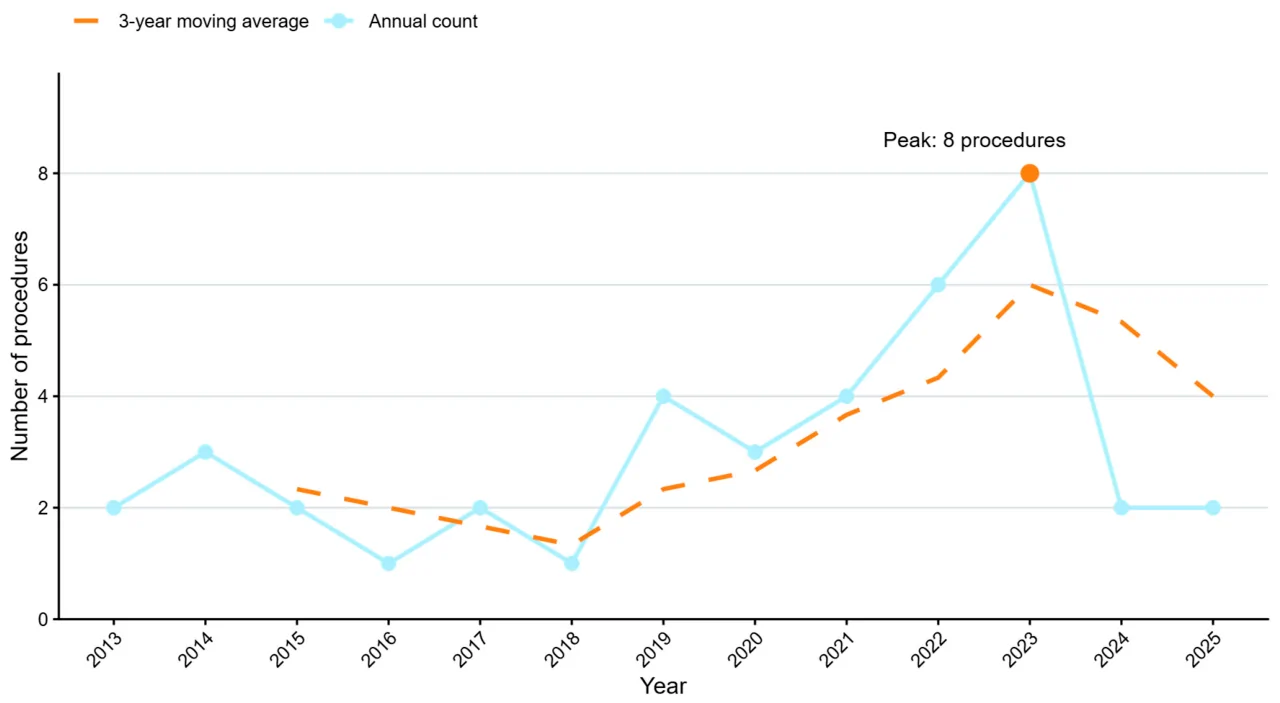
Application of BMDO in infants and neonates in Poland.

The highest annual number of MDO procedures was recorded in 2023, when eight procedures were performed, accounting for 20.0% of the entire cohort. Overall, 18/40 procedures (45.0%) were performed between 2021 and 2023. Figure 2 illustrates the annual number of infants and neonates who underwent a BMDO procedure in Poland between 2013 and 2025.

### 3.3. Preoperative Respiratory Support

All patients included in the study cohort presented with respiratory insufficiency. Thirty-seven patients required some form of respiratory support. Conservative airway management, including prone or lateral positioning, was employed in 10/40 patients (25.0%); 27/40 (67.5%) required device-assisted respiratory support, and 18/40 (45.0%) underwent airway instrumentation before BMDO.

Positioning therapy was used in 8/23 (34.8%) iPRS patients compared with 2/17 (11.8%) sPRS patients. Device-assisted respiratory support was required in 14/23 (60.9%) iPRS patients and 13/17 (76.5%) sPRS patients (*p* = 0.333). Preoperative airway instrumentation was recorded in 9/23 (39.1%) iPRS patients and 9/17 (52.9%) sPRS patients (*p* = 0.523).

Prior to mandibular distraction osteogenesis, tracheostomy had been performed in 10 patients. It was substantially more frequent among patients with sPRS than those with iPRS: 7/17 (41.2%) versus 3/23 (13.0%), respectively. This difference demonstrated a strong trend but did not reach the conventional threshold for statistical significance (*p* = 0.066). Detailed characteristics of the subgroups are presented in Table 1.

### 3.4. Overall Airway Outcome and Tracheostomy Avoidance

A successful airway outcome was defined according to preoperative airway status: tracheostomy avoidance, without the need for further respiratory support in non-tracheostomized patients (assessed at the end of activation phase), or successful decannulation, with no requirement for additional respiratory support in patients with pre-existing tracheostomy (decannulation up to 12 months since surgery).

According to this definition, a successful airway outcome was achieved in 30/40 patients (75.0%). Success was observed in 22/23 patients with iPRS (95.7%) compared with 8/17 patients with sPRS (47.1%). This difference was statistically significant (*p* < 0.001).

Patients with a successful overall airway outcome were significantly younger than those in whom respiratory success was not achieved. Median age was 54 days (IQR 35.0–69.8) in the successful group compared with 138 days (IQR 78.3–176.5) in the unsuccessful group (*p* = 0.015).

Patients with a pre-existing tracheostomy were significantly older at MDO than those without a tracheostomy. Their median age was 144.5 days (IQR 105.8–208.0) compared with 49.5 days (IQR 34.3–68.0) among non-tracheostomized patients (*p* < 0.001). Age was not associated with the requirement for device-assisted respiratory support (*p* = 0.897) or preoperative airway instrumentation (*p* = 0.654).

Thirty patients did not have a tracheostomy before MDO and therefore constituted the population at risk of requiring a new postoperative tracheostomy. Of these, 28/30 (93.3%) avoided tracheostomy following distraction. All 20 initially non-tracheostomized patients with iPRS remained free of postoperative tracheostomy, corresponding to a tracheostomy-avoidance rate of 100% (20/20). In contrast, 8/10 initially non-tracheostomized patients with sPRS avoided tracheostomy, corresponding to a rate of 80.0%. Two patients in the sPRS group required a new postoperative tracheostomy. The difference between iPRS and sPRS did not reach statistical significance because of the small number of events (*p* = 0.103). Considering the entire cohort without accounting for preoperative tracheostomy status, postoperative tracheostomy was recorded in 2/40 patients (5.0%), both of whom belonged to the sPRS group (0/23 [0%] iPRS vs. 2/17 [11.8%] sPRS; *p* = 0.174).

Among the 10 patients who had a tracheostomy before MDO, seven patients presented with sPRS and three presented iPRS. Successful decannulation was documented in 2/10 (20.0%). Both successful decannulations occurred among patients with iPRS. Thus, 2/3 (66.7%) previously tracheostomized iPRS patients were successfully decannulated, compared with 0/7 (0%) patients with sPRS. Despite the marked numerical difference, statistical significance was not reached because of the very small subgroup size (*p* = 0.067). Table 2 presents airway outcomes following mandibular distraction osteogenesis.

### 3.5. Hospitalization Length

The median total hospital stay was 36 days (IQR 25.5–44.0) among patients with iPRS and 23 days (IQR 6–43) among patients with sPRS. The mean total hospital stay for the whole cohort was 33.4 days (±22.0). There was no significant difference between groups (*p* = 0.411).

A strong inverse correlation was observed between patient age at the time of surgery and total length of hospitalization (Spearman’s ρ = −0.655, *p* < 0.001), indicating shorter hospital stays among older patients. This association remained statistically significant when the preoperative and postoperative hospitalization periods were evaluated separately (ρ = −0.405, *p* = 0.009 and ρ = −0.609, *p* < 0.001, respectively).

Considering the postoperative intensive care unit, 29/40 patients needed ICU care postoperatively. The median ICU stay was 9 days (IQR 6.0–10.5) in the iPRS group, 17 days (IQR 7.0–19.5) in the sPRS group and 11.2 days (±7.6) overall. ICU stay was significantly longer among patients with sPRS (*p* = 0.049).

In contrast to the findings for overall hospitalization, no statistically significant association was identified between patient age at surgery and the duration of postoperative ICU stay (ρ = −0.186, *p* = 0.333).

**Figure 3:**
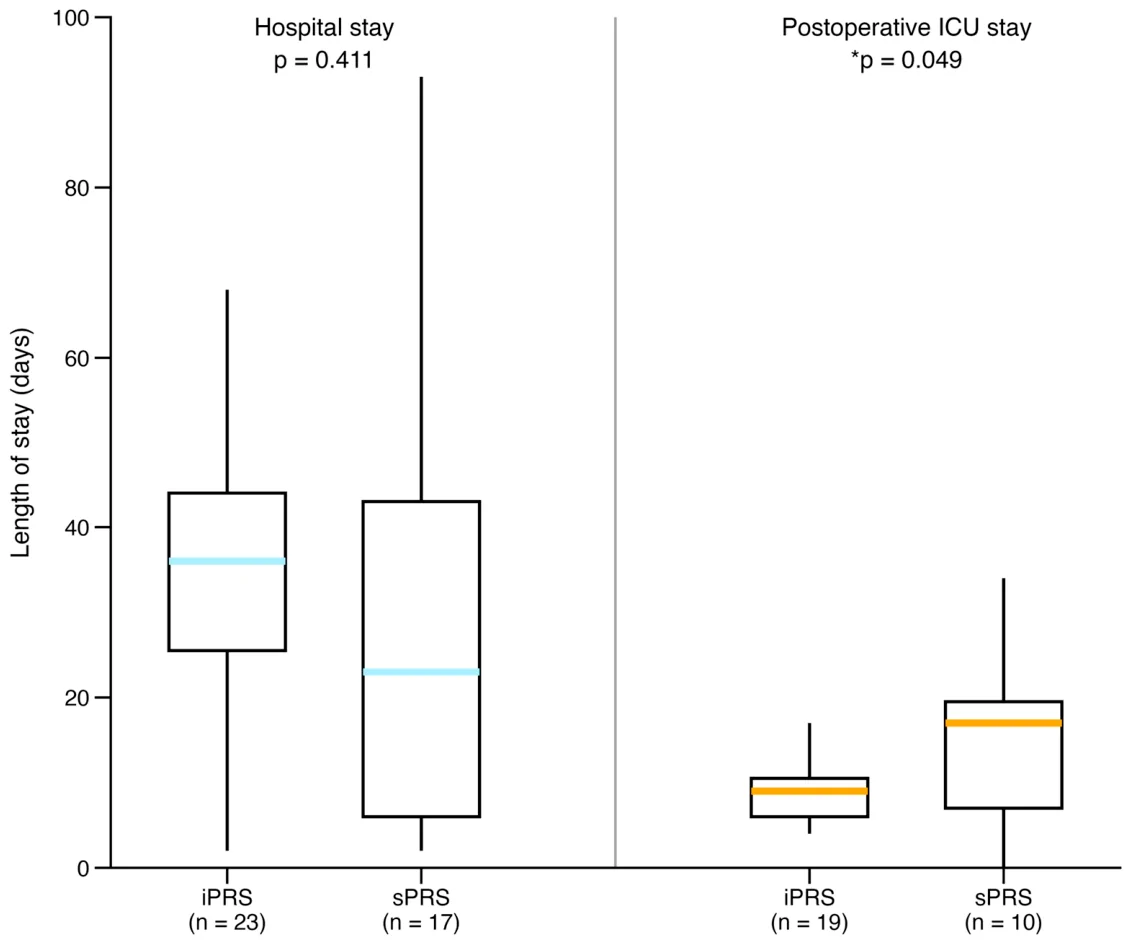
Hospital stay and ICU stay in iPRS and sPRS subgroups. n = number of patients; iPRS—isolated Pierre Robin sequence; sPRS—syndromic Pierre Robin sequence; * *p* < 0.05 in Mann—Whitney U test.

Hospital and ICU stay details are presented in Figure 3.

### 3.6. Multivariable Analysis of Respiratory Outcome

In the study cohort 30 of 40 patients (75.0%) achieved respiratory success whereas 10 patients (25.0%) met the criteria for respiratory failure. Syndromic status (sPRS) remained independently associated with respiratory failure after adjustment for age in the primary model. The first model included syndromic Pierre Robin sequence (sPRS) status and age at mandibular distraction osteogenesis (MDO). Patients with sPRS had significantly higher odds of respiratory failure compared with those with isolated Pierre Robin sequence (adjusted odds ratio [aOR] 12.11; 95% CI 2.21–123.02; *p* = 0.003). Age at MDO was not independently associated with respiratory outcome after adjustment for syndromic status (aOR 1.056 per week; 95% CI 0.983–1.144; *p* = 0.140). Table 3 presents results of univariable regression.

**Figure 4:**
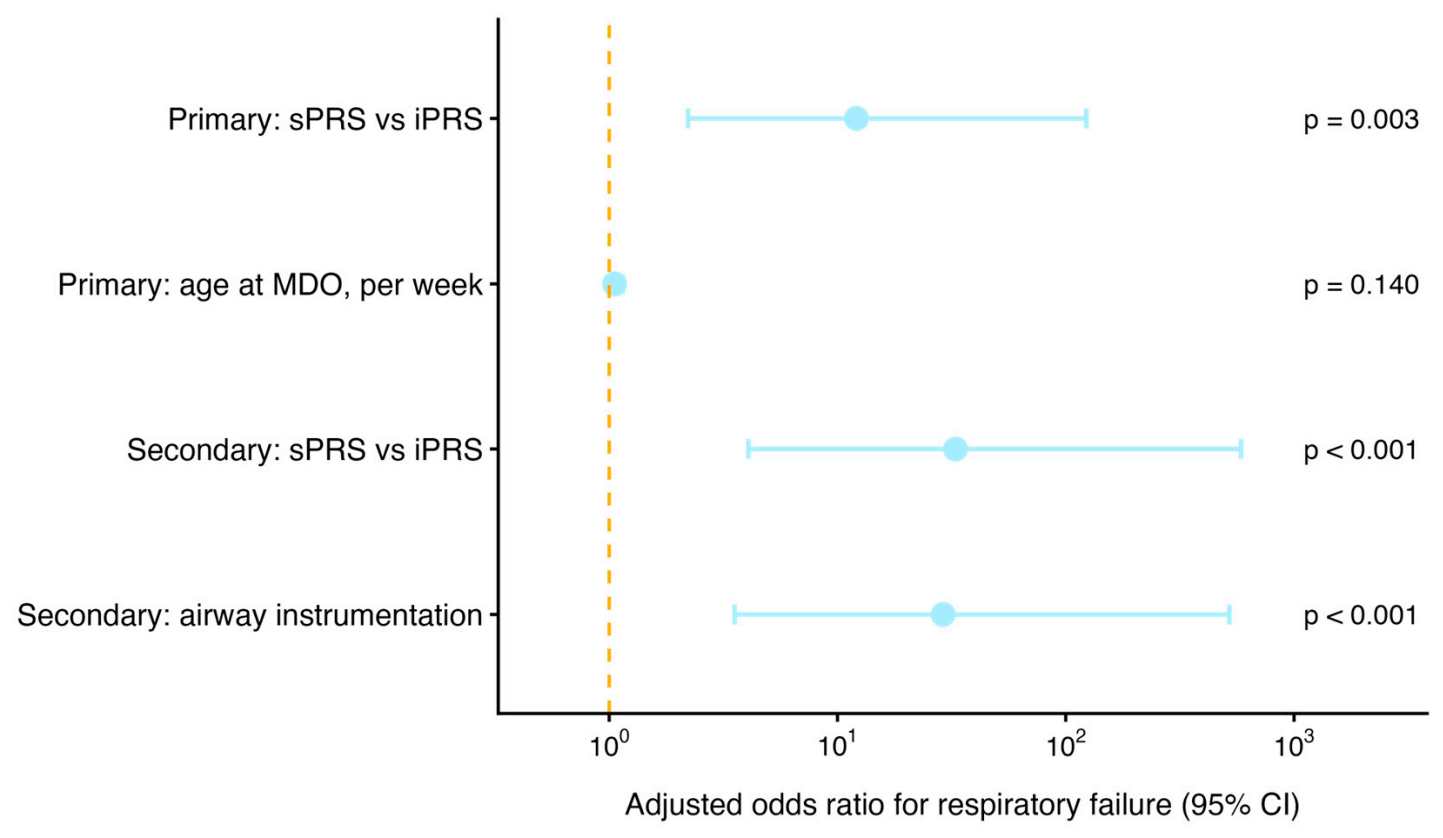
Adjusted associations from the primary and secondary models. CI—confidence interval.

In a secondary exploratory model including sPRS status and preoperative airway instrumentation, both sPRS (aOR 32.88; 95% CI 4.08–586.28; *p* < 0.001) and preoperative airway instrumentation (aOR 29.01; 95% CI 3.54–521.38; *p* < 0.001) were significantly associated with respiratory failure. Similar findings were observed in a three-variable exploratory model including sPRS, age at MDO and preoperative airway instrumentation. In this model, sPRS (aOR 20.04; 95% CI 2.56–323.54; *p* = 0.003) and preoperative airway instrumentation (aOR 21.26; 95% CI 2.73–371.39; *p* = 0.002) remained significant whereas age at MDO did not (aOR 1.050 per week; 95% CI 0.946–1.173; *p* = 0.371). Associations of primary and secondary models are presented in Figure 4.

## 4. Discussion

This study presents the first analysis of airway outcomes following BMDO in PRS patients in the Polish population. The principal findings were that BMDO provided effective airway management, with an overall successful airway outcome achieved in 75.0% of patients. A successful airway outcome was achieved in 95.7% of patients with iPRS, compared with 47.1% of those with sPRS. Furthermore, BMDO was highly effective in preventing a new tracheostomy in patients who were not tracheostomy-dependent before distraction. Successful decannulation was considerably less frequent among patients with a pre-existing tracheostomy.

The temporal distribution of BMDO procedures in our cohort demonstrates a gradual increase in the use of mandibular distraction over the study period, with a particularly marked rise in the number of procedures performed after 2020. Importantly, epidemiological data on the incidence and prevalence of Pierre Robin sequence in the Polish population are currently lacking. It is difficult to determine whether the increasing number of procedures reflects a true increase in the number of affected patients or changes in referral and treatment patterns. PRS epidemiology was described in population-based EUROCAT research which reported PRS prevalence from 1998 to 2017. The study surveyed a total of 1294 cases of PRS in Europe. The overall prevalence was 12.0 per 100,000 births. The prevalence was higher in the most recent 10-year period (2008–2017), possibly due to better perinatal diagnosis. The researchers report that the 68.2% of cases constituted isolated PRS and 19.3% were correlated with congenital anomalies out of which 6% were genetic syndromes. The male-to-female ratio was 0.94. One of the registered participating centers was located in Wielkopolska, Poland. During the whole time of the observation 51 cases of PRS were registered there. Out of these 51 cases, 37 (72.5%) cases were isolated PRS [4]. In our study, the isolated PRS constituted 57.5% of the study group and the sPRS was 42.5% [15]. Therefore, the researchers can not cite any representative epidemiological data for the Polish population. These values cannot be interpreted as a reflection of the Polish population as the hospital treats the most severe cases. According to the European survey on current practice patterns in PRS treatment, BMDO accounts for 33% of procedures [16].

The mean age during operation in our group was 12.4 (±11 weeks) while in the systematic review on distraction osteogenesis in PRS by Dominguez et al. the mean age for BMDO was 100 days. Children in our center were operated on average 13 days earlier than in the analyzed studies [17]. This could be dictated by the fact that our center is the only referential center in Poland where surgical treatment of children with congenital anomalies takes place. Moreover, physicians across the country are aware of the necessity of fast-track treatment for these patients. Taking into consideration the influence of age in two subgroups, patients with iPRS were significantly younger at the time of mandibular distraction osteogenesis than those with sPRS, with a median age of 52 days compared with 97 days. According to the study by Scott et al. the mean age at the time of the intervention was 4.8 weeks. They reported 19 patients of whom 14 presented with iPRS and five were diagnosed with sPRS, which is consistent with our study [18]. In comparison the study conducted by Goza et al. reports 28 patients with PRS with a mean age of 135 days at the time of the procedure [19]. The study conducted by Kolstad et al. reports that the older the patient at the time of operation, the poorer the respiratory outcome [20]. The publications reporting TCS patients who underwent neonatal MDO are less frequent and report BMDO at a significantly older age than in iPRS patients [21,22]. These papers show differences between two groups and confirm the difficulties in treating the syndromic cases. The older age at the time of distraction in the sPRS group may also indicate a greater need for early airway management in these patients, particularly in cases of severe upper airway obstruction associated with micrognathia and glossoptosis.

A particularly important finding of the present study is the distinction between tracheostomy avoidance and decannulation. Among the 30 patients who did not have a tracheostomy before BMDO, 28 (93.3%) remained free of tracheostomy following distraction. The outcome was particularly favorable in iPRS, in which all 20 initially non-tracheostomized patients avoided subsequent tracheostomy. In the corresponding sPRS subgroup, eight of 10 patients avoided tracheostomy, while two required a new postoperative tracheostomy. The overall tracheostomy-avoidance rate demonstrates that BMDO can provide effective airway management. The clinical situation was markedly different in patients who were already tracheostomy-dependent before BMDO. Only two of the 10 previously tracheostomized patients were successfully decannulated following distraction. Both successful decannulations occurred in patients with iPRS, corresponding to a decannulation rate of 66.7% in previously tracheostomized iPRS patients, whereas none of the seven tracheostomy-dependent patients with sPRS achieved decannulation. Our findings correspond with those presented in other studies. They concluded that BMDO was found to be 95% successful at preventing tracheostomy. Among patients who were tracheostomy-dependent before the procedure, the decannulation was less frequent. The failure rate was even greater in sPRS [23,24,25,26].

The difference in the airway outcome between iPRS and sPRS emphasizes the importance of the underlying diagnosis. Successful airway management was achieved in 22/23 patients with iPRS compared with only 8/17 patients with sPRS (95.7% vs. 47.1%, *p* < 0.001). Our finding is in line with others which demonstrate that patients with syndromic craniofacial disorders constitute a substantial proportion of those with unfavorable outcomes and were also more frequently tracheostomized before BMDO [27,28]. Accordingly, the diagnosis of sPRS may identify a clinically more complex population in whom correction of mandibular hypoplasia alone is less consistently associated with complete airway independence [23,28,29].

Hospitalization data provide additional information regarding the perioperative burden associated with BMDO. Total hospital stay did not differ significantly between diagnostic groups, with median durations of 36 days in iPRS and 23 days in sPRS. In contrast, postoperative ICU stay was significantly longer in patients with sPRS, with a median of 17 days compared with 9 days in iPRS. This finding is consistent with the generally more complex postoperative course observed in the syndromic subgroup [30,31].

The multivariable analysis suggests that syndromic status was the most consistent baseline factor associated with respiratory failure after BMDO. Although older age at distraction was associated with failure in univariable analysis, this relationship was no longer significant after adjustment for sPRS status. It indicates that the apparent effect of age may have been at least partly confounded by the higher proportion of older and clinically more complex patients within the syndromic group. In contrast, the association between sPRS and respiratory failure persisted across all multivariable models. It supports the concept that the underlying syndromic diagnosis may be a more important determinant of treatment outcome than age at distraction alone.

Preoperative airway instrumentation also demonstrated a strong independent association with respiratory failure in exploratory models. This finding likely reflects greater baseline airway compromise and overall clinical severity rather than a direct causal effect of airway instrumentation itself. Importantly, the very wide confidence intervals observed for both sPRS and preoperative airway instrumentation indicate substantial uncertainty in the magnitude of these associations. Such uncertainty is expected given the small sample size and the occurrence of only 10 respiratory failures. Accordingly, these models should be interpreted as exploratory rather than definitive predictive models. Nevertheless, the consistency of the association between sPRS and respiratory failure, together with the strong signal related to preoperative airway severity, suggests that syndromic diagnosis and baseline respiratory status may be useful factors for identifying patients at increased risk of an unfavorable respiratory outcome following BMDO.

Several limitations of this study should be acknowledged. First of all, the study was based on a relatively small cohort of 40 patients. This may limit the statistical power of subgroup analyses, particularly those involving the syndromic PRS group and patients with preoperative tracheostomy. Secondly, the retrospective nature of the study introduces the potential for selection and information bias. Moreover, the sPRS group comprised a heterogeneous range of underlying syndromes, each potentially associated with distinct craniofacial anatomy, airway abnormalities, comorbidities, and postoperative courses. The multivariable analysis is at risk of bias because of only 10 events in the presented cohort. Consequently, the results for the syndromic group should not be interpreted as representative of a single disease phenotype. These limitations should be considered when interpreting the observed associations and when comparing the findings with those of larger, prospective studies.

## 5. Conclusions

To conclude, BMDO was associated with a high rate of successful overall airway outcomes. Seventy-five percent of patients achieved either avoidance of postoperative tracheostomy or successful decannulation. Airway success was significantly more frequent among patients with iPRS than among those with sPRS. This suggests that the underlying clinical phenotype may be an important determinant of airway outcome following distraction. Patients with iPRS also underwent BMDO at a significantly younger age than those with sPRS. Patients with sPRS may not be the best candidates for the early BMDO treatment because of preexisting modalities. Patients with preoperative tracheostomy underwent BMDO at a significantly older age and demonstrated lower rates of successful decannulation, particularly within the syndromic subgroup. sPRS is an unrelated diagnostic factor of BMDO failure in the multivariable analysis.

These findings confirm BMDO to be an effective procedure for achieving airway improvement and avoiding or reversing tracheostomy in appropriately selected patients with PRS. Larger prospective studies with standardized indications, longer follow-up, and syndrome-specific analyses are warranted to better define predictors of airway success and long-term surgical burden.

## Figures and Tables

**Table 1:** Baseline characteristics and preoperative respiratory status according to diagnostic group.

Variable	iPRS (n = 23)	sPRS (n = 17)	*p*
Median of Age at MDO, days, (IQR)	52 (35–63)	97 (43–153)	0.030 *
Male sex, n (%)	11 (47.8)	14 (82.4)	0.046 †
Device-assisted respiratory support, n (%)	14 (60.9)	13 (76.5)	0.333
Preoperative airway instrumentation, n (%)	9 (39.1)	9 (52.9)	0.523
Preoperative tracheostomy, n (%)	3 (13.0)	7 (41.2)	0.066

* *p* < 0.05 in Mann–Whitney U test; † *p* < 0.05 in Fisher’s exact test.

**Table 2:** Airway outcomes following mandibular distraction osteogenesis.

Outcome	Overall	iPRS	sPRS	*p*
Tracheostomy avoidance in patients without preoperative tracheostomy	28/30 (93.3%)	20/20 (100%)	8/10 (80.0%)	0.103
Successful decannulation in patients with preoperative tracheostomy	2/10 (20.0%)	2/3 (66.7%)	0/7 (0%)	0.067
New postoperative tracheostomy, entire cohort	2/40 (5.0%)	0/23 (0%)	2/17 (11.8%)	0.174
Composite successful airway outcome *	30/40 (75.0%)	22/23 (95.7%)	8/17 (47.1%)	<0.001 †

† *p* < 0.05 in Fisher’s exact test. * Defined as tracheostomy avoidance, without the need for further respiratory support in non-tracheostomized patients (assessed at the end of activation phase), or successful decannulation, with no requirement for additional respiratory support in patients with pre-existing tracheostomy (decannulation up to 12 months since surgery).

**Table 3:** Univariable Firth penalized logistic regression for respiratory failure after MDO.

Predictor	OR	95% CI	*p* Value
sPRS vs. iPRS	16.76	3.14–174.39	<0.001 *
Age at MDO, weeks	1.075	1.012–1.158	0.018 *
Gestational age, weeks	0.89	0.66–1.19	0.402
Prematurity (<37 weeks)	1.46	0.29–6.49	0.626
Male sex	5.57	1.07–56.47	0.040 *
Cleft palate	0.19	0.03–1.14	0.069 *
Preoperative airway instrumentation	14.33	2.71–147.97	<0.001 *
Preoperative tracheostomy *	38.76	6.75–338.08	<0.001 *

MDO, mandibular distraction osteogenesis; iPRS, isolated Pierre Robin sequence; sPRS, syndromic Pierre Robin sequence; OR, odds ratio; CI, confidence interval; * *p* < 0.20 in Firth penalized logistic regression.

## Data Availability

Publicly available data are provided within the manuscript. Data which are unavailable due to privacy or ethical restrictions can be requested from P.K.
